# M1 Macrophage‐Derived Extracellular Particles Induce Cell Death in MDA‐MB‐231 Cells

**DOI:** 10.1002/cnr2.70237

**Published:** 2025-07-02

**Authors:** Parth Desai, Anjali Kumari, Saqer Al Abdullah, Azreen Anwar, Kyle Nowlin, Kristen Dellinger

**Affiliations:** ^1^ Department of Nanoscience, Joint School of Nanoscience and Nanoengineering University of North Carolina at Greensboro Greensboro North Carolina USA; ^2^ Department of Nanoengineering, Joint School of Nanoscience and Nanoengineering North Carolina A&T State University Greensboro North Carolina USA; ^3^ Department of Psychology and Neuroscience University of North Carolina at Chapel Hill Chapel Hill North Carolina USA

**Keywords:** caspase, cytotoxicity, extracellular particles, macrophage, triple‐negative breast cancer

## Abstract

**Background:**

Triple‐negative breast cancer (TNBC), a leading cause of female mortality worldwide, presents a treatment challenge due to the lack of targeted receptors. Macrophages, recognized for their role in the immune response, provide a promising avenue for cancer research. Given that macrophages secrete extracellular particles (EPs), which have been implicated in biological processes, including intercellular communication and immune modulation, it is hypothesized that EPs derived from macrophages could have potential anticancer effects.

**Aims:**

This study examines the effect of M1 macrophage‐secreted EPs on TNBC cells to investigate their potential as a therapeutic.

**Methods and Results:**

Polarization was induced in RAW 264.7 macrophages and characterized using ELISA, nitrite release, and microscopy. Macrophage‐derived EPs were isolated and characterized using nanoparticle tracking analysis, electron microscopy, and western blotting. The influence of EPs on MDA‐MB‐231 cells, a TNBC model, was assessed using confocal microscopy. Results showed the increasing expression of caspase 3/7 in a time‐dependent manner (0, 24, and 48 h). Cell death was observed in TNBC cells with M1 macrophage‐derived EPs, while cell proliferation was observed when M2 macrophage‐derived EPs interacted with MDA‐MB‐231 cells.

**Conclusion:**

Overall, results showed that EPs derived from M1 macrophages could induce cell death in MDA‐MB‐321 cells, opening up potential options for new treatments in TNBC.

## 1 Introduction

Breast cancer is the leading cause of death in women worldwide [1, 2]. Among the different types of breast cancer, they are named according to their respective receptor presence, namely human epidermal growth factor receptor (HER) 2 positive, progesterone receptor (PR) positive, estrogen receptor (ER) positive breast cancer, and triple‐negative breast cancer (TNBC). TNBC is the most aggressive type of breast cancer, and it is the leading cause of death in females aged 20–59 due to its metastatic nature [3]. According to Gogate et al., the number of metastatic breast cancer cases is predicted to increase by 54.8% by 2030 in the United States [4]. Currently, pembrolizumab is the only cancer immunotherapy approved by the FDA to treat early‐stage TNBC [5]. To meet the demand of these increasing metastatic breast cancer cases, there is a need to develop more effective and safe therapies that can help overcome TNBC [4].

Extracellular particles (EPs) are nanosized multimolecular bodies secreted by various cell types in the body that include but are not limited to extracellular vesicles (EVs) or vesicle‐like structures, according to the recent guidelines of the International Society of Extracellular Vesicles (ISEV) [6, 7]. The role of EPs is variable, though they are often considered cargo carriers that mediate cell‐to‐cell communication [8]. EPs are composed of vesicular and non‐vesicular structures derived from the cells, and due to their natural ability to encapsulate and carry cellular materials like nucleic acids, proteins, chemokines, and cytokines, they can be leveraged as a potential candidate for cancer therapy. The role of EPs in TNBC therapeutics is a topic of debate [9], especially since small EVs derived from TNBC cells can create an immunosuppressive environment by attacking critical immune cells like T cells and inducing apoptosis [10, 11]. They have also been shown to affect the differentiation of monocytes into dendritic cells (DCs) and induce myeloid‐derived suppressor cells (MDSCs) [12, 13]. Immature MDSCs have been indicated to help tumors evade immune surveillance and make different cancer treatments ineffective [14, 15]. On the other hand, small EVs derived from various immune cells, such as DCs, natural killer cells (NK cells), T cells, and B cells have shown anti‐cancer or pro‐tumorigenic properties [16].

Tumor progression has been linked with chronic inflammation and dysregulated activity of immune cells [17] and is supported by a complex tumor microenvironment composed of different immune cells, extracellular matrix, non‐immune cells, and vascular structures. Macrophages, a central cell type in tumor biology and immunology, play a crucial role in tumor progression or inhibition based on the signaling molecules they receive from the tumor microenvironment [18, 19]. Tumor‐derived EVs have been shown to change the fate of macrophage polarization, which determines the tumor‐inhibitory or tumor‐promoting effect of macrophage cells [18]. Researchers have found that breast cancer‐derived EVs can influence macrophages by regulating different pathways to support the tumor microenvironment [17, 20, 21]. Generally, macrophages polarized in the M1 phenotype (classically activated) indicate tissue inflammation, and the M2 phenotype (alternatively activated) creates an anti‐inflammatory environment. The M1 phenotype is characterized by increased inducible nitric oxide (iNOS) synthase, an anti‐tumorigenic and inflammatory marker, while the M2 phenotype is more of a wound‐healing phenotype supporting tissue growth, cell migration, and metastasis via upregulation of arginase‐1 and CD‐206 molecules [22, 23]. Other papers have also detailed the role of tumor‐associated M2 macrophages, which have been associated with poor prognosis, chemoresistance, and metastasis [24, 25]. EVs derived from M1 macrophages have been shown to exhibit the ability to transport a diverse array of chemokines, cytokines, and cellular proteins, demonstrating a potentially promising avenue for advancing TNBC treatment strategies [26, 27, 28].

For example, Baek et al. developed effective PEGylated M1 macrophage‐derived exosome mimetic nanovesicles (MNVs) to increase the accumulation of MNVs in the tumor. Their research showed M1 macrophage‐derived exosome mimetic nanovesicles enhanced cancer‐targeting ability in a CT 26 tumor model [29]. Moreover, studies have demonstrated that when 4T1, a breast cancer tumor model, was targeted with docetaxel‐loaded M1‐derived EVs, it prompted the transformation of M0 macrophage phenotypes into the M1 phenotype within the tumor microenvironment [30]. In another study, M1 macrophage‐derived exosomes were targeted against chemoresistance in pancreatic cancer. The exosomes loaded with gemcitabine and deferasirox offer an excellent combination for therapeutic applications to potentially overcome chemoresistance [31]. In addition to these studies, paclitaxel‐loaded M1 macrophages also showed the inhibition of 4T1 breast cancer cells [32].

**FIGURE 1:**
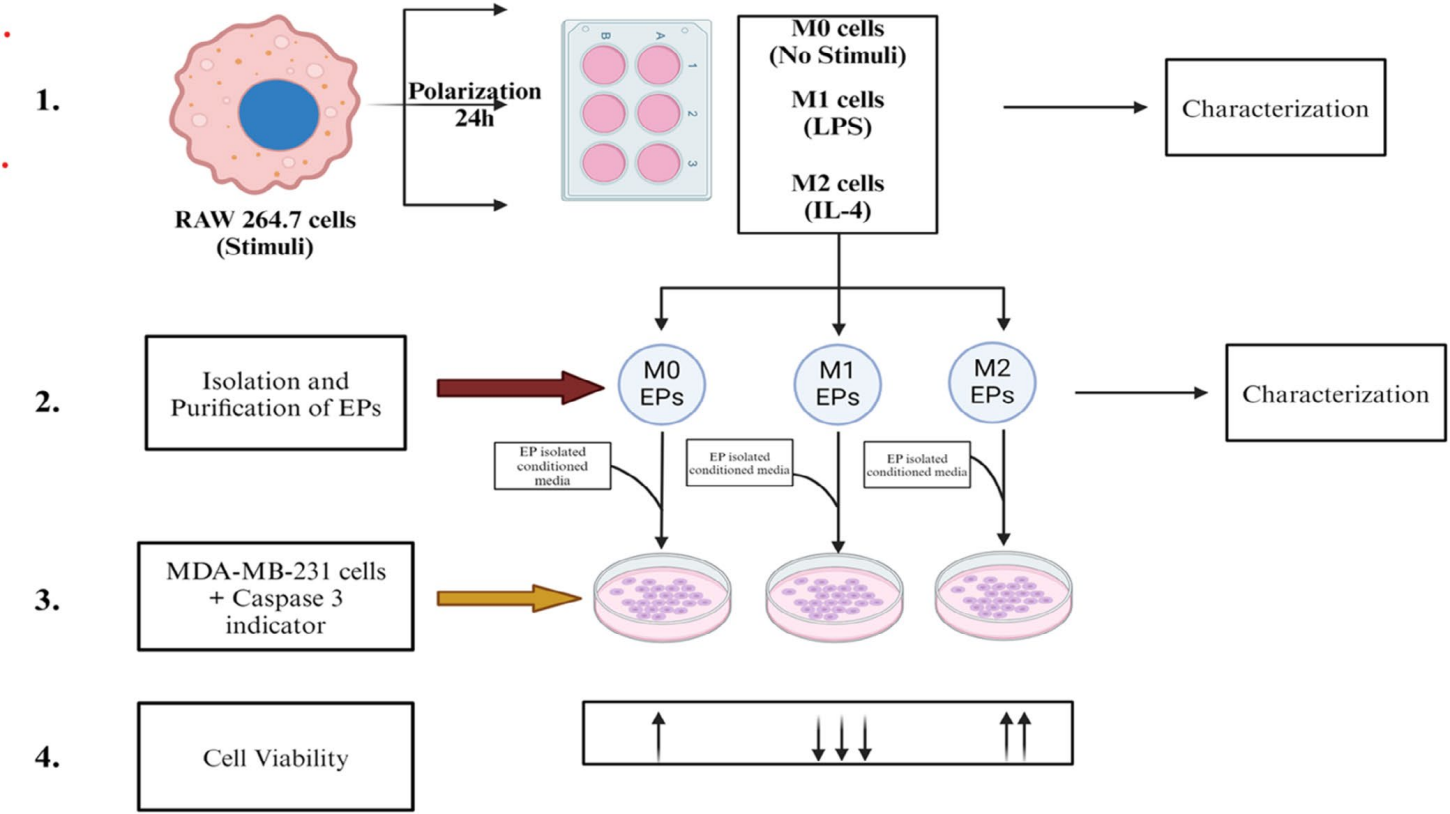
Experimental design schematic: 1. RAW 264.7 cells were grown in a 6‐well plate to compare M0, M1, and M2 stages. The cells' M1 and M2 states were polarized using lipopolysaccharides (LPS) and interleukin (IL‐4) as stimuli, respectively. The cells and collected cell culture media were subjected to characterization of polarization markers. 2. RAW 264.7 cells were cultured in conditioned media that was fetal bovine serum (FBS)‐depleted to isolate and purify EPs from collected cell culture media in M0, M1, and M2 polarized states. All EPs were characterized after isolation and purification. 3. Isolated EPs were tested against MDA‐MB‐231 cells and caspase 3/7. EP‐isolated conditioned media was added to MDA‐MB‐231 cells to differentiate between cell culture media effects from M0, M1, and M2 RAW 264.7 cells with M0, M1, and M2 isolated EPs. 4. An XTT assay was performed to measure cell viability. The up arrows (↑) indicate increased proliferation and (↓) indicate decreased proliferation. Created with BioRender.com.

To leverage the available literature and address current challenges in TNBC therapies, we aimed to explore the effect of M0, M1, and M2 macrophage‐derived EPs on TNBC. Specifically, this study aimed to explore the potential effects of macrophage‐derived EPs on MDA‐MB‐231 cells. Based on literature showing EVs exchange between cell types in the tumor microenvironment [16], we hypothesized that if cancer cell‐derived EVs can trigger M2 macrophages to support breast cancer tumor growth, we can use macrophage‐derived EPs to check their effect against TNBC. To test our hypothesis, EPs were isolated from RAW 264.7 cells stimulated with lipopolysaccharide (LPS) or interleukin‐4 (IL‐4) and categorized into M0, M1, and M2 macrophages (Figure 1).

## 2 Materials and Methods

### 2.1 Cell Culture

RAW 264.7 macrophages (ATCC, USA) were cultured using Dulbecco's Modified Eagle Medium DMEM (Gibco, Cat #10569010) with 1% L‐glutamine (Gibco, Cat #25030081) and 10% heat‐inactivated FBS (Gibco, Cat #16140071) at 37°C and 5% CO_2_. MDA‐MB‐231 cells (ATCC, USA) were cultured using Dulbecco's Modified Eagle Medium DMEM (Gibco, Cat #10569010) with 1% L‐glutamine (Gibco, Cat #25030081) and 10% heat‐inactivated FBS (Gibco, Cat #16140071) at 37°C and 5% CO_2_. The cells were monitored and passaged when they reached 80% confluency.

### 2.2 Macrophage Polarization Morphological Analysis

RAW 264.7 cells were seeded at a density of 4 × 10^5^ cells per well in 6‐well plates to induce polarization into M1 and M2 phenotypes using lipopolysaccharide (LPS) (Thermo Fisher, USA) and mouse interleukin 4 (IL‐4) (eBioscience, Cat#BMS338), respectively. Polarization was induced based on a previously developed protocol [33]. Briefly, RAW 264.7 cells were stimulated with LPS and IL‐4 at the following concentrations: 0, 25, 50, and 100 ng/mL. Macrophages were incubated with inducing agents for 24 h, and polarization was verified under an Olympus IX51 bright‐field microscope. Images were taken using the SeBaView software and an external camera.

### 2.3 Biochemical Analysis of Macrophage Polarization

To verify RAW 264.7 cells polarization, cells were seeded at a density of 4 × 10^5^ per well in a 6‐well plate. The levels of pro‐inflammatory markers IL‐6 and TNF‐α were assessed using an IL‐6 mouse ELISA kit (Invitrogen, Cat#KMC0061) and a TNF‐α mouse ELISA kit (Invitrogen, Cat#BMS607‐3). We followed the manufacturer's protocol to perform the assay. All the reagents and standards were prepared at room temperature according to the protocol. Next, 50 μL of cell culture media from M0 uninduced (Control), M1 LPS‐induced, and IL‐4‐induced M2 cells was collected to take the readings, and color change was measured using a multi‐mode microplate reader at a wavelength of 450 nm (Synergy MX).

RAW 264.7 cells at a density of 4 × 10^5^ per well in a 6‐well plate were treated with LPS at different concentrations ranging from 0 to 100 ng/mL. After incubation for 24 h, cell culture media was collected to assess the presence of nitrites using a Griess reagent kit (Invitrogen, Cat#G7921), according to the manufacturer's protocol. Briefly, reagents were combined in a 1:1 ratio and incubated with the samples collected from LPS‐induced RAW 264.7 cells. Nitrite levels were compared among M0 uninduced (Control), LPS‐induced M1 polarized, and IL‐4‐induced M2 polarized cells. Absorbance was determined at 540 nm using a multi‐mode microplate reader (Synergy MX).

### 2.4 EP Isolation and Characterization

#### 2.4.1 EP Isolation

RAW 264.7 cells (8 × 10^6^ cells) were washed three times with 1× sterile PBS and cultured in serum‐free media (SFM) for 3 days in a T75 flask, as described in [34, 35]. After 72 h, media was collected and subjected to centrifugation for 10 min at 1000 rpm, 4°C, and 30 min at 3000 rpm, 4°C to remove cell debris. Per the manufacturer's protocol, the remaining cell culture media was subjected to EP isolation using an ExoQuick‐TC isolation kit (Systems Biosciences, USA). Briefly, the isolation solution was added to the collected cell culture media in a ratio of 1:5 and incubated for 24 h at 4°C. Following this incubation, the solution was centrifuged at 1500 rpm for 30 min at 4°C to pellet the EPs and purified further according to the manufacturer's instructions. Finally, a series of centrifugation and washing steps were followed to yield EPs. According to this study's purification method and characterization, we refer to our isolated vesicles by the generic term “EPs”.

#### 2.4.2 Nanoparticle Tracking Analysis

Isolated EPs were further characterized using nanoparticle tracking analysis (NTA) for particle concentration and size. Approximately 0.3 mL of isolated EPs were loaded into the sample chamber of an LM10 unit (Nanosight, Amesbury, UK), and data analysis was performed with the NTA software (Nanosight). In NTA, the paths of EPs act as point scatterers, undergoing Brownian motion in a chamber through which a 632 nm laser beam is passed. Samples were analyzed using the following parameters: the shutter speed was 15 ms, with camera gains between 280 and 560. Software settings for analysis were detection threshold: 12 multi; blur size: auto; frames per second: 23.75; measurement time: 30 ms. When samples contained higher numbers of particles, they were diluted before analysis, and the relative concentration was calculated according to the dilution factor.

#### 2.4.3 Scanning Electron Microscopy

After purification, EPs were pelleted at 4°C for 100 min at 14000 g and resuspended in a 3.7% glutaraldehyde solution, incubated for 30 min, and re‐centrifuged at 4°C for 100 min. The supernatant was discarded, and EP pellets were washed in 40%, 60%, and 95% ethanol for 15 min each. During the final step, EPs were resuspended in 95% ethanol and deposited on a silicon wafer for SEM analysis. Samples were left overnight to dry under a laminar flow.

MDA‐MB‐231 cells at the density of 4 × 10^5^ per well in a 6‐well plate; M1 RAW 264.7 cells derived EPs with 2 × 10^8^ particles/mL were added to MDA‐MB‐231 cells. After 24 h, the silicon wafer was dipped into 3.7% glutaraldehyde for 30 min. Then, the extra solution was removed and transferred to 40%, 60%, and 95% ethanol. Samples were left to dry overnight before observation under SEM. The interaction between the EPs derived from M1 RAW 264.7 cells and MDA‐MB‐231 cells. Samples were then observed under SEM (JEOL JSM‐IT800 Schottky FESEM).

#### 2.4.4 Western Blot Analysis

Protein levels were detected after isolating EPs from RAW 264.7 cells and characterized by western blot analysis, based on previously published protocols [20, 30, 31]. Briefly, total protein concentration from the samples was measured using a Qubit 4 Fluorometer (Invitrogen, Thermo Fisher). Next, 4× Laemmli buffer was added to samples at a 1:3 ratio and heated to 75°C for 10 min. 19.5 μL of each normalized sample was loaded onto a 12% Mini‐PROTEAN TGX Precast Protein Gel (Bio‐Rad, Hercules, CA). Proteins were separated by electrophoresis, followed by transfer to 0.45 μm nitrocellulose membranes (ThermoFisher). Membranes were blocked with StartingBlock T20 (TBS) blocking buffer for 30 min at 4°C, followed by washing with phosphate buffer saline‐Tween (PBST). RAW 264.7 cell‐derived EP proteins were assessed using anti‐Hsp70 (ab181606, Abcam). Membranes were incubated with primary antibodies (1:1000) overnight at 4°C. Then, membranes were washed 3× for 10 min each in PBST. For primary antibodies, Hsp 70 was incubated with goat anti‐rabbit IgG (A16104, ThermoFisher). Secondary antibodies were diluted in 1:10000, and the membranes were incubated with the secondary antibodies for 30 min at 4°C. The blot was imaged under the iBright Imaging System (Invitrogen, model #FL1500). Each western blot was repeated three times, with the representative images shown in this manuscript.

### 2.5 Confocal Microscopy and Induction of Caspase 3/7

MBA‐MB‐231 cells were stained with BioTracker 405 Blue Mitochondria Dye (Sigma Aldrich, USA, Cat#SCT135). Briefly, MDA‐MB‐231 cells were counted, and the mitochondria tracking dye was added to a final concentration of 40 nM. Cells were incubated at 37°C for 30 min with the dye and seeded in confocal dishes for 24 h. Cells were allowed to attach to the surface of a 4‐chamber confocal dish. The media was replaced the next day with fresh media. Then, EPs isolated from M0 (Control), M1, and M2 polarized RAW 264.7 cells at a concentration of 2 × 10^8^ particles/mL were added to each well in addition to Cell Event Caspase‐3/7 Green ReadyProbes Reagent (Invitrogen, Cat#R37111). Images were taken using an Evident FV3000 confocal microscope using a 20× objective at 0, 24, and 48 h time points.

### 2.6 Cell Viability Studies

A cell viability assay was performed using a CyQUANT XTT (2,3‐bis(2‐methoxy‐4‐nitro‐5‐sulfophenyl)‐2H‐tetrazolium‐5‐carboxanilide) cell viability assay kit (ThermoFisher, USA). Following the manufacturer's protocol, MDA‐MB‐231 at 5000 cells/well were seeded in a 96‐well plate for 24 h. After 24 h, M0 (control), M1, and M2 RAW 264.7 cell‐derived EPs were added at a concentration of 2 × 10^8^ particles/mL to measure cell viability in each sample allowed to incubate. After 24 h incubation with EPs, 1 mL of electron coupling reagent was mixed with 6 mL of XTT reagent and incubated at 37°C for 4 h in a 5% CO_2_ incubator. Absorbance was measured at 450 and 660 nm using a Synergy MX multi‐mode microplate reader. The data analysis was performed according to the manufacturer's protocol.

### 2.7 Statistical Analysis

Statistical analyses were performed using Microsoft Excel. A Student's *t*‐test was employed for *p*‐value determination across all experiments (*N* = 3). All data are expressed as the mean ± SEM (*N* = 3). All raw data readings were exported into Excel, where subsequent calculations were performed, and the resulting data were plotted.

## 3 Results

### 3.1 Macrophage Polarization Morphological Differences

**FIGURE 2:**
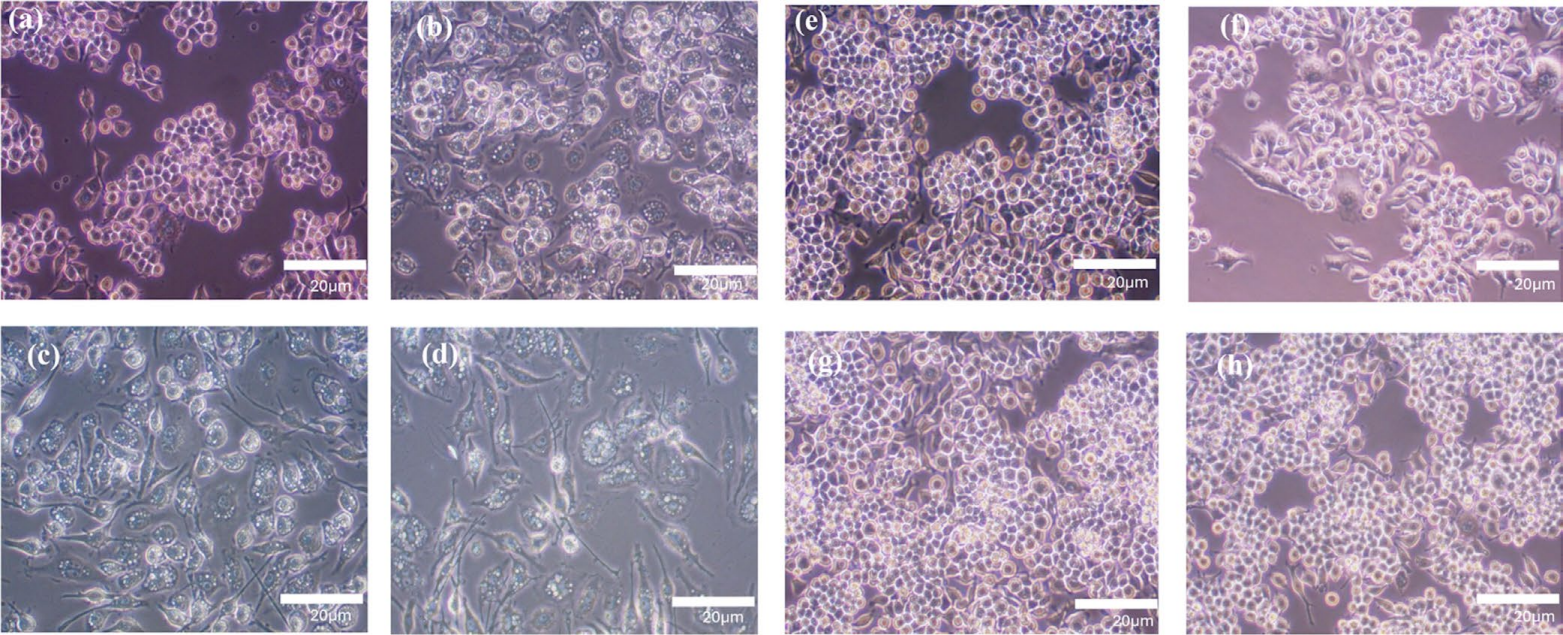
RAW 264.7 cells under the influence of different stimuli: RAW 264.7 cells were observed for M1 polarization in the presence of LPS after 24 h (a) 0, (b) 25, (c) 50, and (d) 100 ng/mL. M2 polarization was observed in the presence of IL‐4 after 24 h (e) 0, (f) 25, (g) 50, and (h) 100 ng/mL. The observation was made using a bright field microscope with a 10× objective and an external camera; images were collected using SeBaView software.

Macrophages are innate immune cells characterized by their functional characteristics and environmental responses, known as M0 (resting/not activated), M1 (classically activated), or M2 (alternatively active). Lipopolysaccharide (LPS) is an inducer of the M1 macrophage phenotype, while IL‐4 is an inducer of the M2 macrophage phenotype [33, 36]. Accordingly, we used different concentrations of LPS to polarize RAW 264.7 cells and monitored their morphology via optical microscopy. After 24 h, round cells were observed in the non‐induced (M0 macrophages) RAW 264.7 cells, the control group (Figure 2a), while RAW 264.7 cells with LPS (M1 macrophages) looked like dendritic or star‐like shapes (Figure 2b–d), indicating a morphological change. (Figure 2f–h) indicate insignificant changes in shape under the influence of IL‐4 (M2 macrophages) compared to non‐induced RAW 264.7 cells (Figure 2e), the control group. Thus, macrophage polarization was determined morphologically after 24 h under external stimuli of LPS and IL‐4 under different concentrations (0–100 ng/mL). We found that LPS‐induced (M1 macrophages) RAW 264.7 cells showed noticeable differences in morphology compared to non‐induced cells (M0 macrophages) in Figure 2a, while IL‐4‐induced (M2 macrophages) RAW 264.7 cells showed insignificant morphological differences compared to non‐induced (M0 macrophages) cells in Figure 2e.

### 3.2 M1 Macrophages Produce Pro‐Inflammatory Markers

**FIGURE 3:**
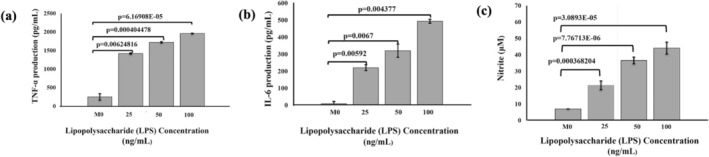
RAW 264.7 cell polarization using biochemical analysis: RAW 264.7 cells were cultured in 6‐well plates and incubated for 24 h before collecting cell culture media. Collected media was subjected to enzyme‐linked immunosorbent assay (ELISA), (a) and (b) show the dose‐dependent increase in the levels of IL‐6 and TNF‐α cytokines. (c) Shows the dose‐dependent increase in the levels of nitrites. Results indicate that M0 polarized RAW 264.7 cells, the control, have low nitrites produced compared to LPS‐induced macrophages. *N* = 3 independent experiments. Error bars = SEM; connecting bars denote the statistical comparison between groups, with *p‐*value < 0.05 considered statistically significant.

LPS‐induced M1 macrophages have been shown to secrete markers, such as tumor necrosis factor‐α (TNF‐α), nitric oxide (NO), and interleukin‐6 (IL‐6), indicating their pro‐inflammatory state [37]. RAW 264.7 cells were challenged with LPS to induce an inflammatory or M1 polarized state. Subsequently, TNF‐α, nitrite production, and IL‐6 levels released by cells were quantified by collecting cell culture media. Results showed that LPS‐induced TNF‐α, nitrite, and IL‐6 concentrations increased in a dose‐dependent manner (Figure 3). TNF‐α and IL‐6 were quantified in cell culture media using ELISA (Figure 3a,b), and the nitrite level was assessed separately in the supernatant using the Griess reaction. We observed the increased nitrite production in a dose‐responsive manner (Figure 3c) compared to M0 or uninduced RAW 264.7 cells. These results confirmed the activated stage of RAW 264.7 cells in a pro‐inflammatory state after LPS induction.

### 3.3 Isolation of EPs and Characterization From M1, M2, and M0 RAW 264.7 Cells

**FIGURE 4:**
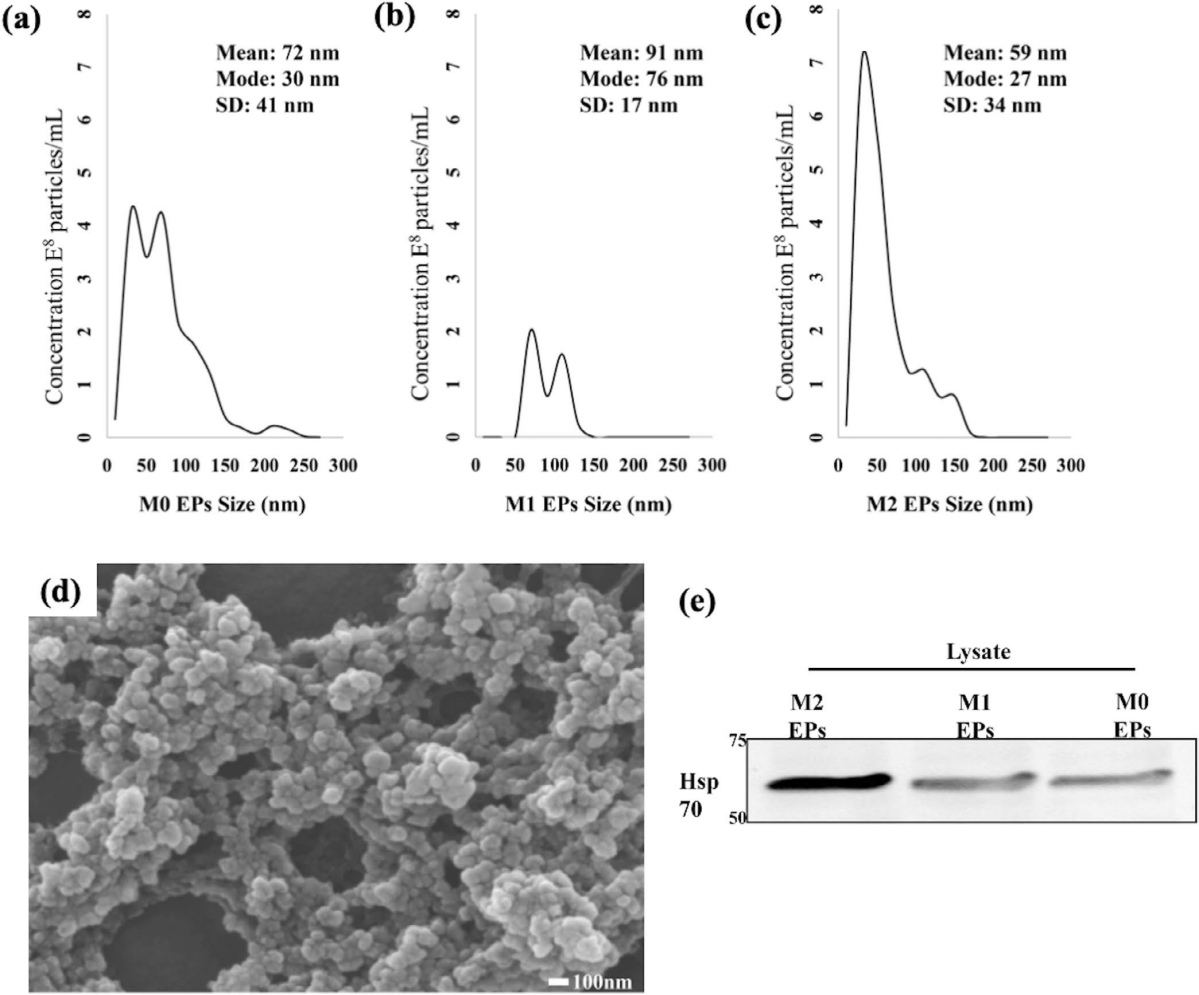
Characterization of EPs isolated from polarized RAW 264.7 cells in their M0, M1, and M2 states: (a–c) particle size distribution showing a heterogeneous population of EPs derived from RAW 264.7 macrophages. The plots show the concentration and particle based on a 15 ms camera shutter speed and 23.75 frames per second per sample type. The data were obtained in the NTA 2.0 analytical software and processed using Microsoft Excel. (d) The SEM image of RAW 264.7 cells derived EPs. EP sizes were consistent with NTA analysis. (e) The presence of HSP 70 was demonstrated with western blotting among all the EPs derived from M0, M1, and M2 polarized RAW 264.7 cells.

Next, EPs derived from three different macrophage states, M0 (uninduced), M1 (induced with LPS), and M2 (induced with IL‐4), were characterized. The characterization was done to identify the type of particle secreted by RAW 264.7 cells under different stimuli. According to the MISEV guidelines [6, 7], EP size was analyzed by NTA and the mode size found among the samples was 27–76 nm, regardless of their polarization state. In addition to their size, it was also observed that the number of particles varies with M0, M1, and M2 polarized states (Figure 4a–c). A scanning electron microscope (SEM) was used to confirm their size and morphology. The EPs had a heterogeneous size range on the order of 100 nm (Figure 4d). Next, protein content in isolated EPs was analyzed using western blot analysis (Figure 4e). After confirming enrichment of heat shock protein 70 (HSP 70), isolated particles from RAW 264.7 cells were classified as EPs. Taken together, these results indicate the successful isolation of EPs from RAW 264.7 cells in M1, M2, and M0 polarization states.

### 3.4 M1 Macrophage‐Derived EPs Initiate Caspase 3/7 Activation and Cell Death in MDA‐MB‐231 Cells

**FIGURE 5:**
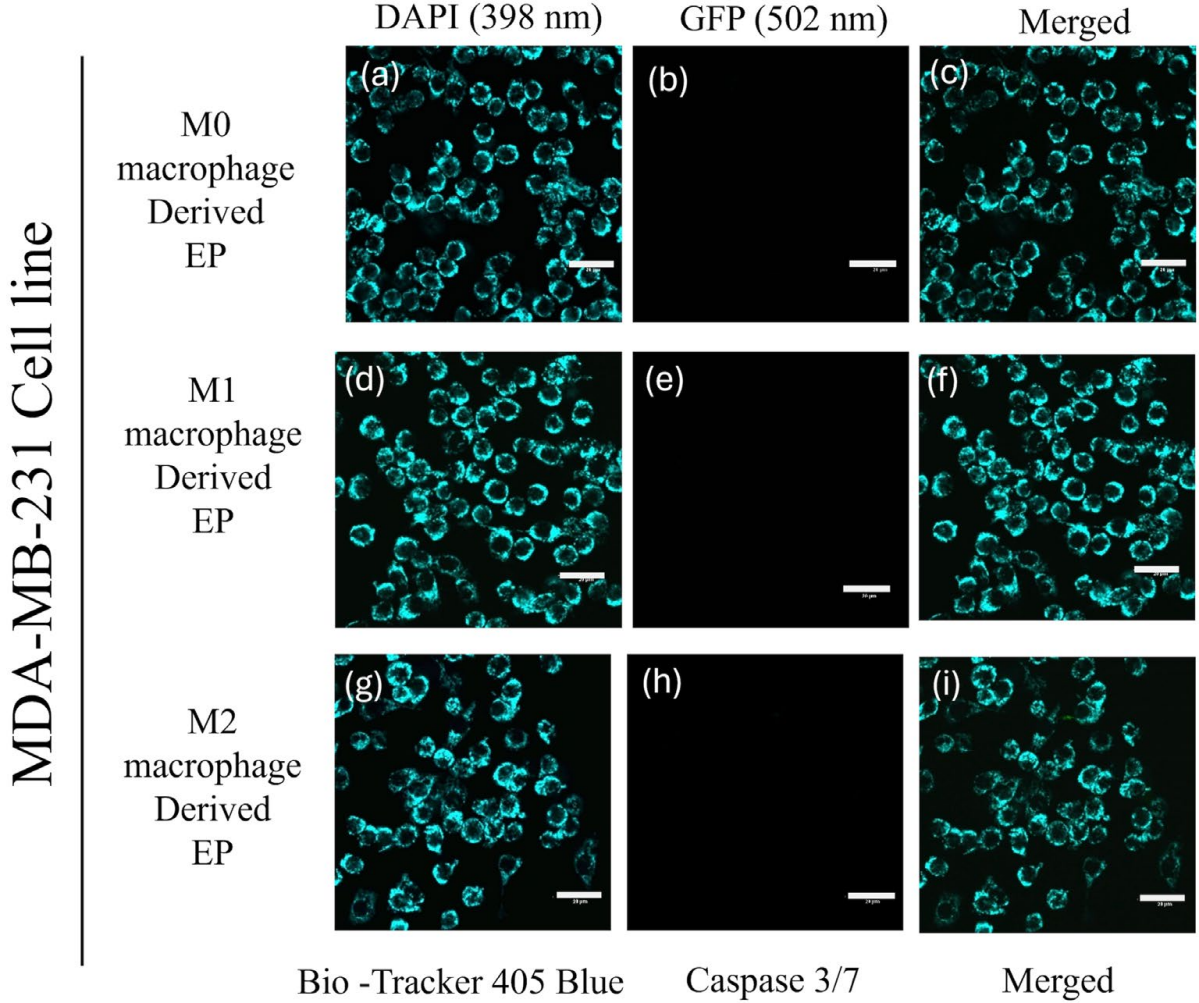
Evaluating caspase‐3/7 expression induced by M1 RAW 264.7 cell‐derived EPs: Confocal images show MDA‐MB‐231 cells at 0 h incubated with EPs derived from M0 macrophages (a–c), M1 macrophages (d–f), and M2 macrophages (g–i). Cells were stained with BioTracker 405 Blue Mitochondrial Dye to visualize mitochondria (a, d, g), while the GFP channel (502 nm excitation) highlights caspase‐3/7 activation (b, e, h). At the 0 h time point, no caspase‐3/7 activation is observed, confirming that the cells are healthy prior to treatment. The scale bar represents 20 μm.

**FIGURE 6:**
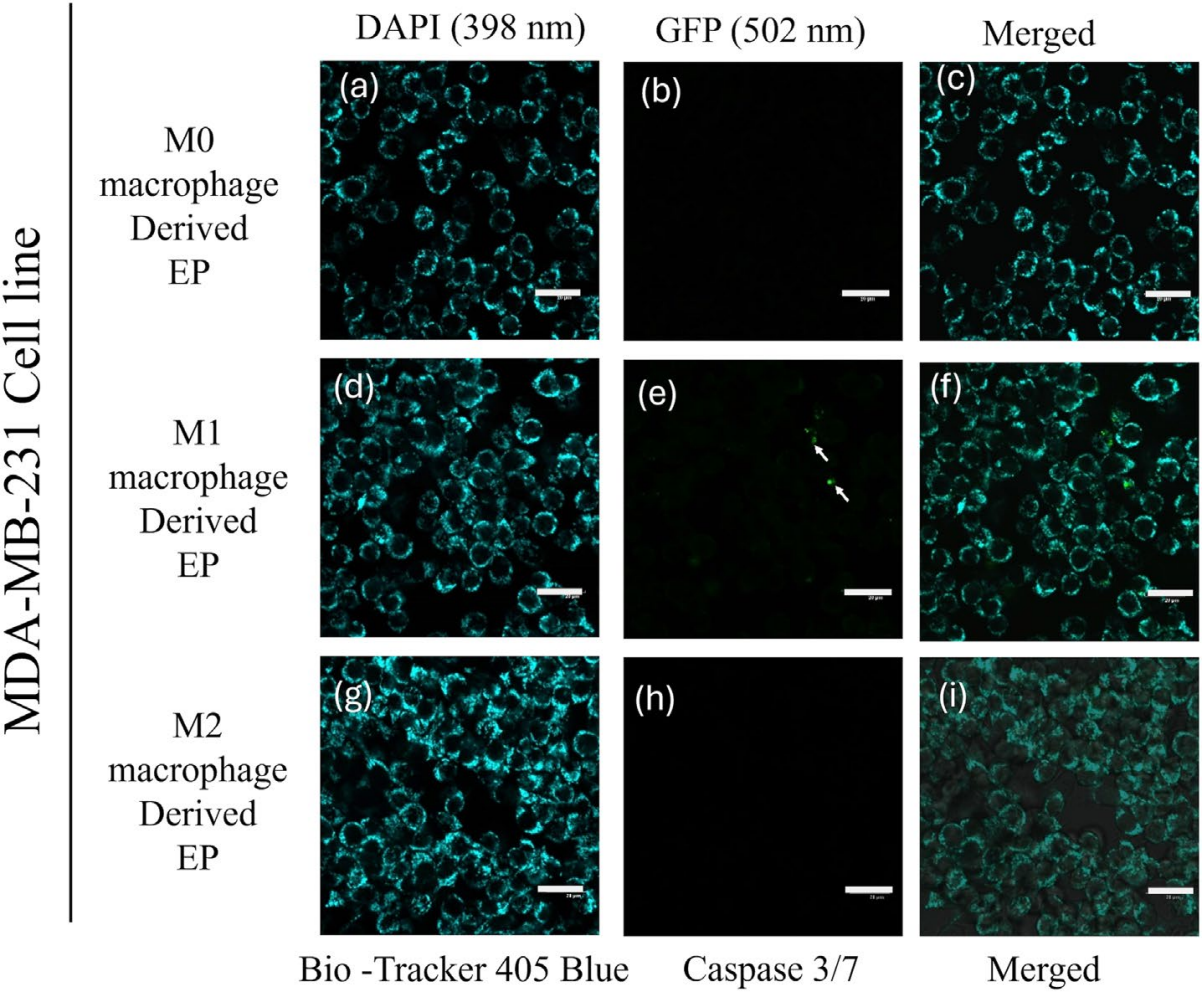
Caspase‐3/7 activation induced by M1 RAW 264.7 cell‐derived EPs at 24 h: Confocal images show MDA‐MB‐231 cells after 24 h of incubation with EPs derived from M0 macrophages (a–c), M1 macrophages (d–f), and M2 macrophages (g–i). Cells were stained with BioTracker 405 Blue Mitochondrial Dye to visualize mitochondria (a, d, g), while the GFP channel (502 nm excitation) highlights caspase‐3/7 activation (b, e, h). At the 24 h time point, caspase‐3/7 activation begins to emerge, indicated by the fluorescence signal in the GFP channel, particularly in cells treated with M1‐derived EPs (e). The scale bar represents 20 μm.

**FIGURE 7:**
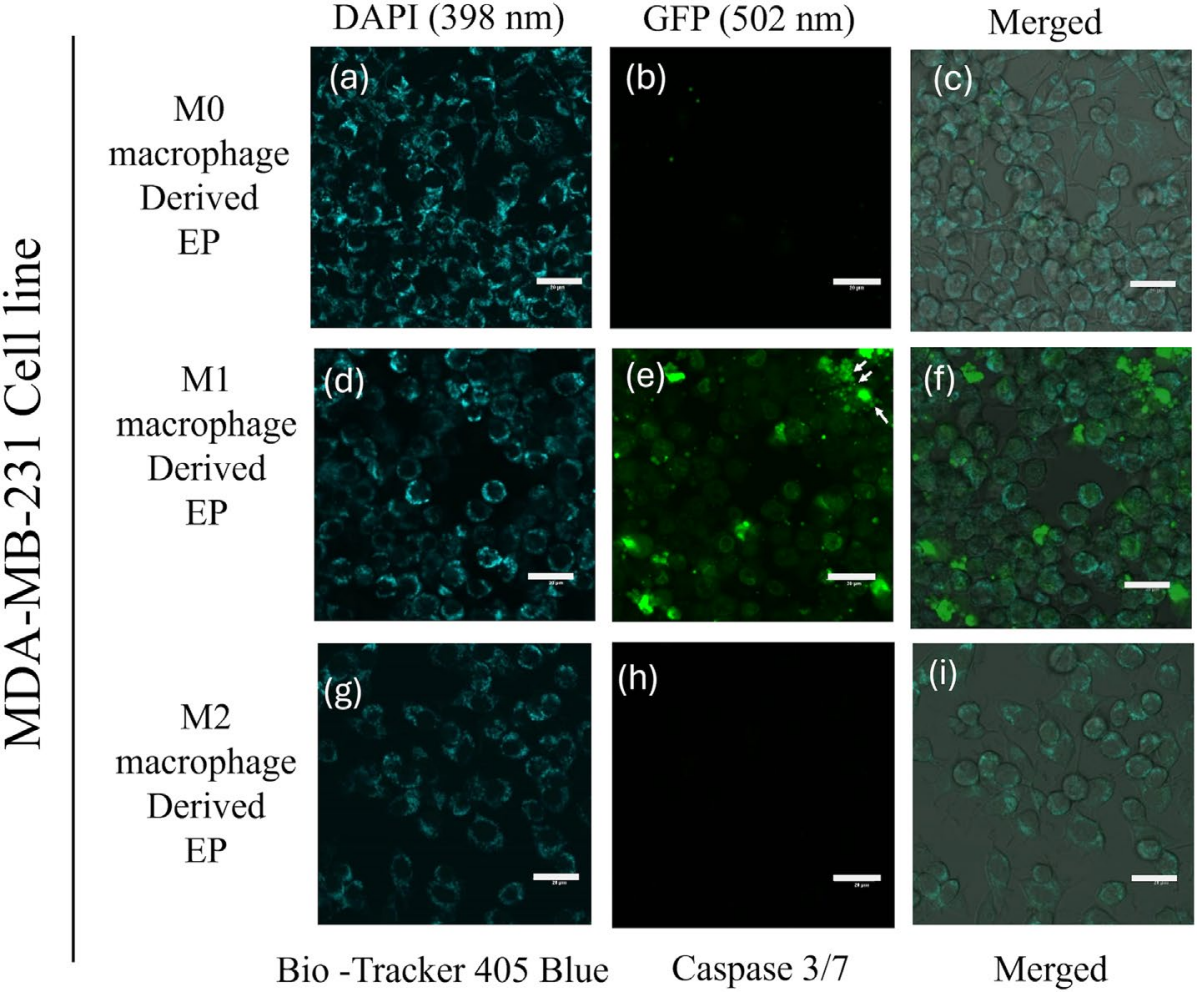
Caspase 3/7 was triggered by M1 RAW 264.7 cell‐derived EPs: The induction of cell death at 48 h in MDA‐MB‐231 cells incubated with M0‐derived EPs (a–c), M1‐derived EPs (d–f), and M2‐derived EPs (g–i). Cells were stained with BioTracker 405 Blue Mitochondrial Dye (a, d, g), and the GFP column at 502 nm excitation (b, e, h) shows the caspase 3/7 activation. The arrows in (e) may indicate cytoplasmic blebs/cell death in small dots around cells. The indicated scale bar is 20 μm.

To evaluate the potency of M0 (Control), M1, and M2 macrophage‐derived EPs on MDA‐MB‐231 cells, EPs were incubated with MDA‐MB‐231 cells for 0 to 48 h. Cells were stained with 405 mitochondrial blue dye and caspase 3/7 dye. Figure 5a–i represents the MDA‐MB‐231 cells at the 0 h time point. The initiation of caspase 3/7 in MDA‐MB‐231 cells was observed after 24 h, Figure 6a–i. At the 48 h time point, Figure 7e shows the dominance of green color indicative of caspase 3/7 activation in MDA‐MB‐231 cells incubated with M1‐derived EPs as compared with other EPs (Figure 7b,h). Figure 5e, taken at 0 h, shows no activity of caspase 3 at the beginning of the experiment. In Figure 6e, the white arrows show the slow induction of caspase 3/7 after 24 h with M1‐derived EPs compared to EPs derived from M0 and M2 RAW 264.7 cells. Possible cytoplasmic blebs indicated by white arrows in Figure 7e were also observed in MDA‐MB‐231 cells after 48 h of incubation with M1‐derived EPs. Thus, these results suggest the possibility of M1‐derived EPs yielding a cytotoxic effect against MDA‐MB‐231 cells after 48 h.

After observing the effects of M1 macrophage‐derived EPs against TNBC, an experiment was performed to investigate interactions via SEM. Samples were prepared using a sterile silicon chip and placed in a 6‐well cell culture plate. After 24 h, EPs were found attaching themselves to MDA‐MB‐231 cells (Figure S1b). Figure S1a shows no morphology change in MDA‐MB‐231 cells when EPs were not added; MDA‐MB‐231 cells clumped together in a ball shape when they were attached to a silicon chip without EPs. While Figure S1b shows cell division, we can differentiate morphological differences in Figure S1a,b. In addition, EPs were observed to be spreading on MDA‐MB‐231 cells, with some potential areas of attachment or anchoring. EPs were observed spreading on MDA‐MB‐231 cells, which seemed to be anchoring to cancer cells (Figure S2b,c, arrows).

### 3.5 Comparing the Cell Viability of MDA‐MB‐231 Cells in the Presence of M0, M1, and M2 EPs

**FIGURE 8:**
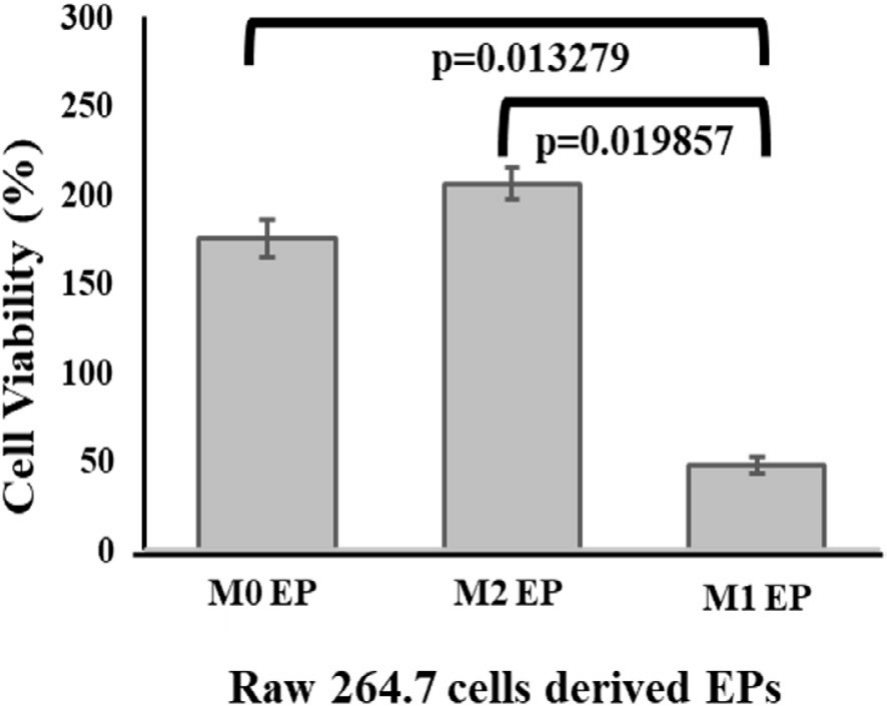
MDA‐MB‐231 XTT cell viability assay: MDA‐MB‐231 cells were exposed to EPs derived from M0 (Control), M1, and M2 RAW 264.7 cells. *N* = 3 independent experiments normalized to the control. Error bars = SEM, connecting bars denote a *p*‐value < 0.05 and were considered statistically significant.

Next, XTT analysis was performed to assess the cytotoxicity of M1‐derived EPs on MDA‐MB‐231 cells. XTT assay results showed only 47% MDA‐MB‐231 cell viability after 24 h of treatment with M1 RAW 264.7 cell‐derived EPs (Figure 8). An MDA‐MB‐231 cell viability of 206% in the sample containing M2 RAW 264.7 cell‐derived EPs was observed, which supports the published literature that suggests that the M2 macrophage‐like phenotype bolsters cancer cell growth [38]. On the other hand, M1‐derived EPs appeared to inhibit the growth of MDA‐MB‐231 cells. Taken together, these results indicate that M1 macrophage‐derived EPs may induce cell death in TNBC cells.

## 4 Discussion

This study indicates the potential for M1 macrophage‐derived EPs to favor tumor interactions [26] and that M1 macrophage‐derived EVs could exhibit anti‐cancer effects. Several studies have explored the potential role of M1 macrophage‐derived EVs' role in TNBC therapy as a potential cytotoxic agent in vitro. For example, macrophage‐derived exosome membranes have been used to load doxorubicin after removing the luminal content and conjugating with PLGA to target c‐Met to show its ability to target TNBC tumors in vivo [39]. However, the effect of EPs derived from macrophages with their native luminal content was never explored. This study identifies and characterize EPs with sizes less than 200 nm in each sample by following MISEV guidelines [6, 7]. A nitrite concentration of 45 μM in collected cell culture media of M1 polarized RAW 264.7 cells was measured [40, 41]. M1 RAW 264.7 cell‐derived EPs with luminal content showed cytotoxicity against MDA‐MB‐231 cells within 48 h by showing time‐dependent increments in caspase‐3/7 expression compared to the M0 uninduced RAW 264.7 cells. This time‐dependent increase in caspase‐3/7 activations in a controlled manner may indicate the EP's ability to release their content in a controlled fashion [42, 43]. Moreover, this study further confirms the ability of M1 RAW 264.7 cell‐derived EPs to decrease cell viability by 53% in 24 h (Figure 8). On the other hand, M2 RAW 264.7 cell‐derived EPs increased the cell viability of MDA‐MB‐231 cells by 206% within 24 h (Figure 8), showing their potential tumor‐supporting effect [22, 23].

In addition, we captured the interaction of EPs with cancer cells that, to our knowledge, have yet to be visualized via SEM. The reason for this anchoring is unclear, but it could be the nature of M1 RAW 264.7 cell‐derived EPs to stimulate the change in morphology of MDA‐MB‐231 cells (Figures S1 and S2). Another reason could be the fusion with the cell membrane of MDA‐MB‐231 cells, which helps EPs internalize or release their content in the MDA‐MB‐231 cell cytosol [7].

## 5 Conclusion

This study revealed that M1 RAW 264.7 cell‐derived EPs can interact with MDA‐MB‐231 cells and induce cell death via caspase 3/7 activation. Anchoring of EPs on TNBC cells has been suggested, which indicates that the other mechanisms of M1 macrophage‐derived EPs could be used to interact with tumor cells [27]. Furthermore, this study paves the way for exploring the content of macrophage‐derived EPs in detail, which can impact the fate of MDA‐MB‐231 cells. In the future, M1 macrophage‐derived EPs could be combined with commercially available therapeutics to characterize the potential for synergistic therapeutic effects [5].

## Author Contributions

**Parth Desai:** conceptualization, methodology, data analysis, writing – original draft. **Anjali Kumari:** data curation, data analysis, methodology. **Saqer Al Abdullah:** data curation, methodology. **Azreen Anwar:** data curation, methodology. **Kyle Nowlin:** data curation, methodology, supervision. **Kristen Dellinger:** conceptualization, writing – review and editing, supervision, funding acquisition.

## Conflicts of Interest

The authors declare no conflicts of interest.

## Supporting information


**Data S1.** Supporting Information.

## Data Availability

The data that support the findings of this study are available from the corresponding author upon reasonable request.
