# Supplementary material for: M1 Macrophage‐Derived Extracellular Particles Induce Cell Death in MDA‐MB‐231 Cells
Source: Cancer Rep (Hoboken). 2025 Jul 2;8(7):e70237. doi: 10.1002/cnr2.70237 (PMC12217045; doi:10.1002/cnr2.70237)
Supplement: Supplementary file 1 — Data S1. Supporting Information. [file CNR2-8-e70237-s001.docx]

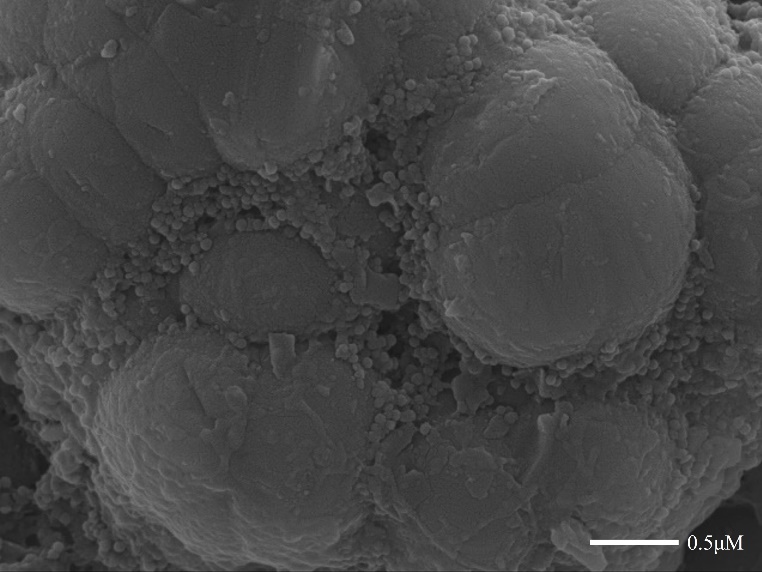


(b)


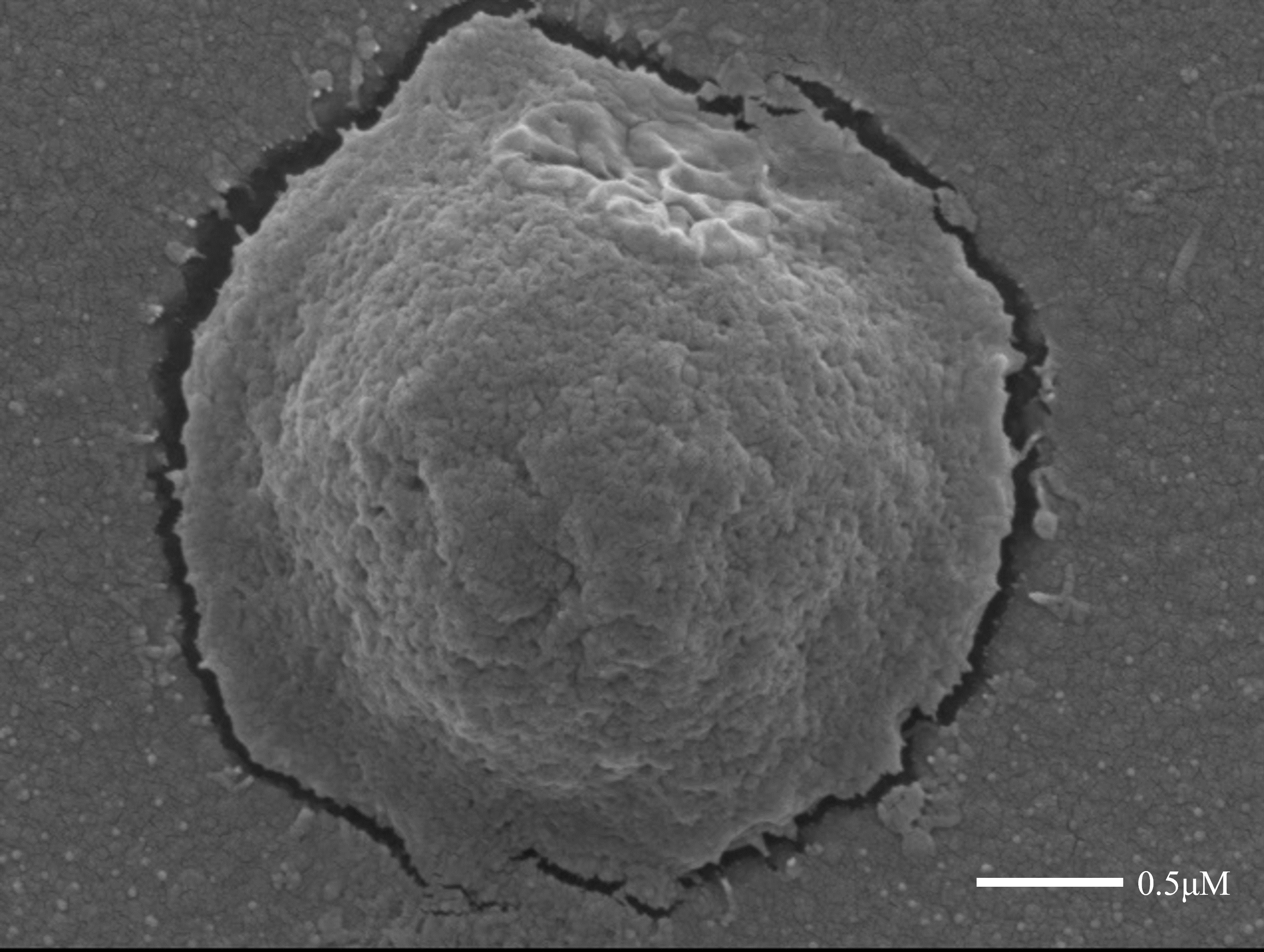


(a)

Figure S1 (A): MDA-MB-231 cells alone without adding EPs to them. After 24h. (B) MDA-MB-231 cells with EPs under the SEM after 24h.

**Figure S2: Evaluating the interaction between M1 RAW 264.7 cell-derived EPs and MDA-MB-231 cells:** (a) shows the morphology of MDA-MB-231 cells when M1 RAW 264.7 cells derived EPs were incubated for 24 h. (b) and (c) White boxes are enlarged in the image, and arrows show the anchored M1 RAW 264.7 cells derived EPs on the MDA-MB-231 cells.


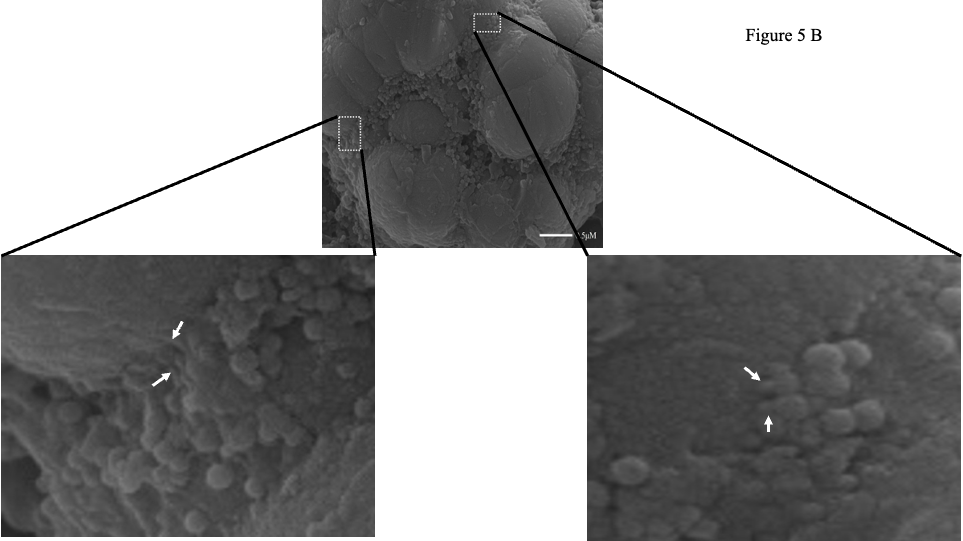


(a)

(b)

(c)
